# The relation between FT_3_ and long-term fatigue in patients with COVID-19

**DOI:** 10.3389/fendo.2024.1411262

**Published:** 2024-08-23

**Authors:** Shuo Dong, FanRui Ding, Yuan Wang, Shuo Liu, Ran Bai, YuanYuan Liu, Yin Zhao, YueRan Zhu, MengXue Liu, Yuenan Liu, Qian Xing

**Affiliations:** Department of Endocrine and Metabolic Diseases, First Affiliated Hospital of Dalian Medical University, Dalian, China

**Keywords:** SARS-CoV-2, COVID-19, thyroid hormone, fatigue, risk factor

## Abstract

**Background:**

Under the current pandemic of Corona Virus Disease 2019 (COVID-19), The relationship between fatigue and COVID-19 has been found. Infection with COVID-19 is associated with fatigue long after the acute phase of COVID-19. Understanding the association of thyroid hormones levels with post-COVID condition, such as fatigue, is necessary to improve quality of life.

**Methods:**

This population-based cohort study was conducted in Dalian, China, from December 2022, to March 2023, using a Yidu Core platform in the First Affiliated Hospital of Dalian Medical University, that integrates medical records, laboratory tests, and all diagnosis and treatment information based on patients in hospital. Eligible individuals were 40 patients with COVID-19, Divided them into fatigue group and non-fatigue group following up by telephone using the FS-14 scale after 6 months. The primary outcomes were the diagnoses of fatigue. The association between thyroid hormones levels and post-COVID condition, such as fatigue, was assessed using logistic regression analysis.

**Results:**

Compared with the non-fatigue group, the FT_3_ level in fatigue group was lower (*p*<0.05). FT_3_ was negatively correlated with fatigue after 6 months (*OR* 0.257, *p*<0.05). After adjusting for confounding factors such as age and gender, low FT_3_ was a risk factor for fatigue in patients with COVID-19, (*OR* 0.225, *p*<0.05). And the FT_3_ is less than 2.47 mol/L, it is the best critical value for predicting long-term fatigue, with a sensitivity of 92.3% and a specificity of 48.1%.

**Conclusions:**

Most people still have fatigue 6 months after COVID-19 infection. FT_3_ serves as the important index to predict fatigue in patients with COVID-19. it should be closely monitored during infection.

## 1 Introduction

The novel coronavirus, which is the cause of the Corona Virus Disease 2019 (COVID-19), has had a significant impact on human life and health. COVID-19 enters host cells to initiate immunity through the binding of angiotensin-converting enzyme 2 (ACE2) or transmembrane protease, serine 2 (TMPRSS2). The high expression of ACE2 and TMPRSS2 in the thyroid gland is even higher than that in lung tissue, so the thyroid gland may be directly affected by COVID-19 (1).

Non-thyroidal illness syndrome (NTIs) are patients without previous basic thyroid diseases. Due to severe systemic diseases, thyroid hormone levels in the blood circulation are abnormally changed. This change is mainly manifested in the decrease of total triiodothyronine (TT_3_) and free triiodothyronine (FT_3_) levels, normal or decreased levels of total thyroxine (TT_4_) and free thyroxine (FT_4_), increased levels of reverse T_3_ (rT_3_), and thyroid stimulating hormone (TSH) levels are in the normal range (2).

It has been observed that many patients diagnosed with COVID-19 often experience abnormal changes in their thyroid hormones, which is known as NTIs. Present, a number of studies have found that FT_3_ levels in patients with COVID-19 are significantly reduced. Zhang et al. found that 28% of COVID-19 patients had thyroid diseases, mainly NTIs (48%) (3), and Łukasz and his colleagues found that COVID-19 patients had a significant decrease in FT_3_ (4). COVID-19 caused changes in thyroid hormones, which may be related to the down-regulation of 5’-deiodinase activity caused by cytokine storm, *in vivo* consumption affecting serum thyroid hormone transporter levels, hypothalamic-pituitary-thyroid axis (HPT) dysfunction and other factors (2). Six months after COVID-19 infection, many people still feel muscle pain, muscle weakness (mild to severe), fatigue and exercise intolerance. Among them, fatigue plays a major role in long COVID syndrome (5).

Currently, there is limited research on the relationship between thyroid hormones and post-COVID syndrome both domestically and internationally. The objective of this study is to investigate the connection between thyroid hormones and long COVID syndrome-fatigue. Additionally, the study aims to identify risk factors that contribute to persistent fatigue following COVID-19 infection. The findings of this research can serve as a guide for the diagnosis, treatment, and comprehensive management of COVID-19 sequelae.

## 2 Methods

### 2.1 Study design and population

Patients with COVID-19 who had no previous or current treatment for hypo- or hyperthyroidism or thyroid surgery history from December in 2022 to March in 2023 in the Department of Endocrinology and Metabolic Diseases, the First Affiliated Hospital of Dalian Medical University were included in the present study. We excluded people with hypothalamic or pituitary disorders, as well as people who were identified to primary neuromuscular or myopathy or who had malignant tumors history. We also excluded individuals who had received radiotherapy or chemotherapy in the past 6 months, and individuals with autoimmune diseases or incomplete data. Moreover, participants who were pregnant or possibly pregnant or ingested agents known to influence thyroid function were also excluded.

The Research Ethics Board at the Dalian Medical University First Affiliated Hospital reviewed and approved this study. All study participants gave their informed consent for participation.

### 2.2 Data source

Data were assembled from the Yidu Core, a large medical management intelligent platform. It contains information on clinical events recorded by health care professionals, including diagnosis, symptoms, and therapies.

### 2.3 Laboratory procedures

COVID-19 infection was defined as nucleic acid test or antigen test was positive. According to the scoring criteria of FS-14, the score ≥ 3 is fatigue, and < 3 is non-fatigue (6–8). The clinical and laboratory data of patients who met the inclusion and exclusion criteria were collected, including gender, age and laboratory examination indicators, such as thyroid stimulating hormone (TSH), serum free triiodothyronine (FT_3_), serum free thyroxine (FT_4_), Alanine aminotransferase (ALT), Aspartate aminotransferase (AST), alkaline phosphatase (ALP), myoglobin (MYO), high-sensitivity troponin I (hs-TnI), creatine kinase isoenzyme (CK-MB), creatine phosphokinase (CK), procalcitonin (PCT), C-reactive protein (CRP), creatinine (Cr), the sum activity of deiodinases (SPINA-GD), the secretory capacity of the thyroid gland (SPINA-GD), TSH index (TSHI), etc. They were followed up by telephone using the FS-14 scale at 6 months after COVID-19 infection. According to their scale scores, they were divided into fatigue group and non-fatigue group.

### 2.4 Biochemical assays

A Mindray automated chemiluminescence immunoassay analyzer CL-6000i (Mindray, China) was used to detect serum TSH, FT_3_ and FT_4_ levels. All test reagents were provided by Shenzhen Mindray Biomedical Electronics Co., Ltd. (Mindray, China). In our laboratory, the reference ranges for TSH, FT_4_. and FT_3_ were 0.35–5.1 uIU/ml, 11.2–23.81 pmol/L, and 2.76-6.45 pmol/L, respectively.

### 2.5 Statistical analysis

Data were analyzed using SPSS 27.0 software. The normality test was performed on the measurement data. Continuous variables are expressed as either the mean – standard deviation or median and interquartile range. T-test or Mann-Whitney U-test was used for comparison between the two groups. Spearman method was used for correlation analysis between index groups, and bilateral test was used. The statistical description of the count data was expressed as [n (%)], and the chi-square test was used for comparison. Binary Logistic regression analysis was used to analyze the risk factors of fatigue. The receiver operating characteristic curve (ROC) and the area under the curve (AUC) were used to compare the cut-off points and predictive values. *p*<0.05 was deemed to show statistical significance.

## 3 Results

### 3.1 Participant demographics

**Figure 1:**
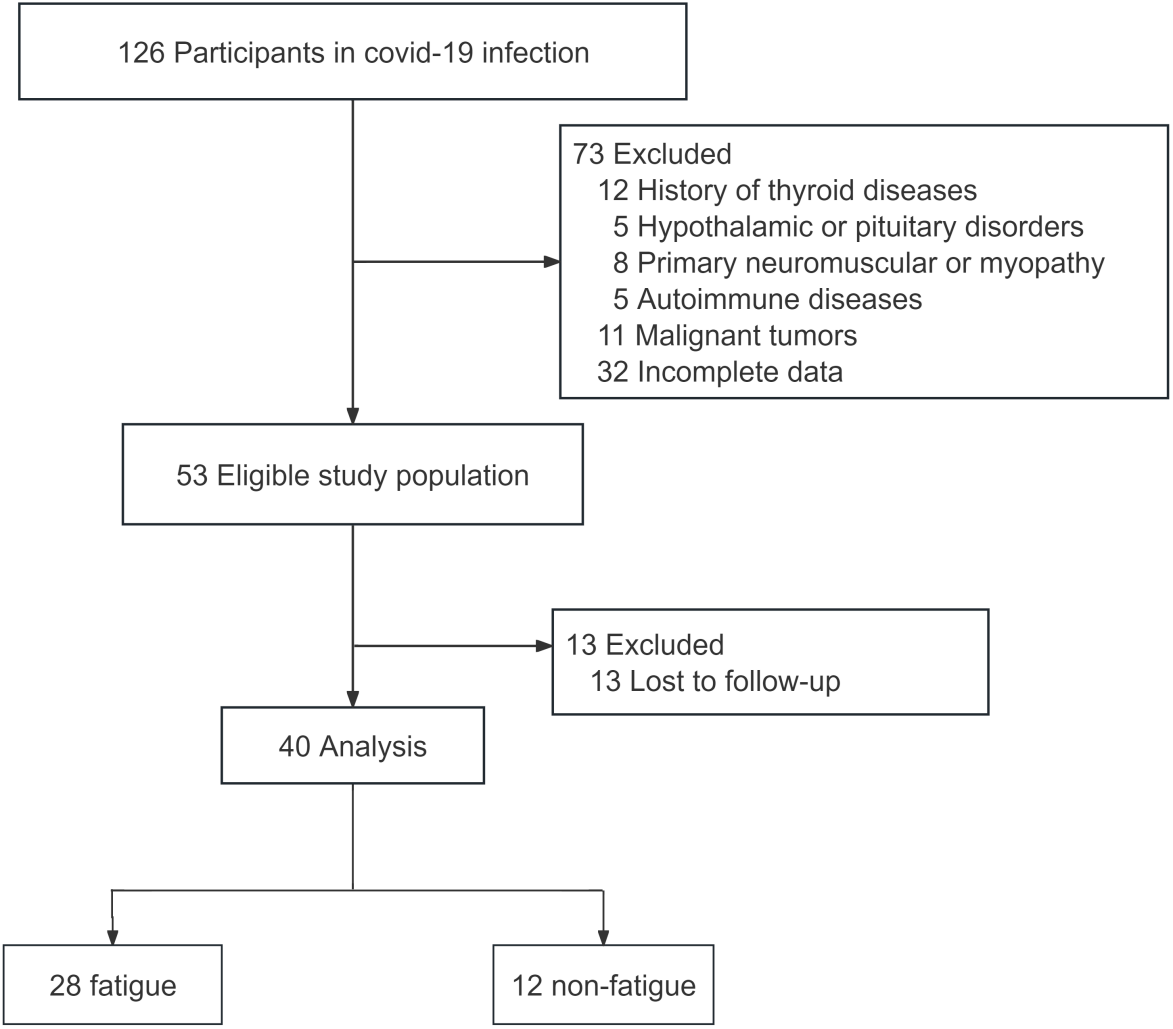
Flow chart of research and analysis of eligible COVID-19 infection participants.

In this study, a total of 126 patients infected with COVID-19 were recruited. After applying the inclusion and exclusion criteria, 40 patients were selected for the study. These patients were followed up by telephone six months after being infected with COVID-19 (**Figure 1**). Based on their fatigue scores, they were divided into a fatigue group and a non-fatigue group. The results showed that there was no statistically difference in sex ratio and age between the two groups (*p*>0.05). The FT_3_ level in the fatigue group was significantly lower than that in the non-fatigue group (*p*<0.05). The difference in CK, TSH, FT_4_, FT_3_/FT_4_ levels, SPINA-GD, SPINA-GT, TSHi was not statistically significant (**Table 1**).

**Table 1:** Demographic, clinical and biochemical characteristics of the study population.

	Fatigue(n=28)	non-fatigue(n=12)	*p*
Age year	72.00(50.00-80.00)	64.00(55.50-78.00)	0.654
Sex Female (n%)	13(33%)	7(59%)	0.739
TSH(μIU/ml)	1.37(0.60-2.43)	1.06(0.73-1.63)	0.444
FT_3_(pmol/L)	2.52 ± 0.63	3.00 ± 0.56	0.023*
FT_4_(pmol/L)	15.91 ± 3.53	16.36 ± 1.15	0.552
FT_3_/FT_4_	0.16 ± 0.035	0.18 ± 0.041	0.160
CK(U/L)	64.00(37.50-145.50)	46.00(20.00-183.50)	0.569
ALT(U/L)	21.00(19.00-43.00)	21.00(13.50-46.00)	0.783
AST(U/L)	28.00(21.00-42.00)	29.00(17.50-42.00)	0.874
ALP(U/L)	81.00(68.00-124.00)	74.00(49.50-100.00)	0.260
MYO(ng/ml)	71.17(34.07-176.43)	47.06(27.09-319.02)	0.638
hs-TnI(μg/ml)	0.016(0.006-0.037)	0.006(0.0055-0.0185)	0.080
CK-MB(μg/ml)	0.92(0.48-1.97)	0.92(0.37-2.33)	0.931
BNP(ng/L)	82.04(47.48-191.18)	50.51(27.70-125.97)	0.128
P(mmol/L)	1.06 ± 0.47	1.10 ± 0.45	0.806
Ca(mmol/L)	2.06(1.96-2.23)	2.08(1.975-2.195)	0.897
PCT(ng/ml)	0.19(0.10-0.52)	0.20(0.02-0.37)	0.754
CRP(mg/L)	13.59(5.33-74.63)	25.10(8.34-33.00)	0.529
fatigue scores	7.00(5.00-9.00)	1.00(0.00-1.50)	<0.001*
SPINA-GD	14.67(12.85-19.81)	17.19(13.87-19.82)	0.069
TSHI	-1.97(-3.11–0.40)	-2.43(-2.91–1.59)	0.453
SPINA-GT	5.06 ± 3.77	5.49 ± 3.38	0.733

TSH, thyroid stimulating hormone; FT_3_, serum free triiodothyronine; FT_4_, serum free thyroxine; ALT, alanine aminotransferase, AST, aspartate aminotransferase; ALP, alkaline phosphatase; MYO, myoglobin; hs-TnI, high-sensitivity troponin; CK-MB, creatine kinase isoenzyme; Ca, Calcium; CK, creatine phosphokinase; P, phosphorus; PCT, procalcitonin; CRP; c-reactive protein; SPINA-GD, the sum activity of deiodinases; SPINA-GT, the secretory capacity of the thyroid gland; TSHI, thyroid stimulating hormone index.

*Level of significance p<0.05.

### 3.2 Association between thyroid hormone and 6-month fatigue in COVID-19

Correlation analysis showed that fatigue scores were significantly negatively correlated with FT_3_/FT_4_ and SPINA-GD levels (*r*=-0.329, *p*<0.05), and fatigue scores had no significantly correlation with Ca, P, CK, and CK-MB (*p*<0.05) (**Table 2**). FT_3_/FT_4_ levels were significantly negatively correlated with hs-TnI (*r*=-0.409, *p*<0.05). FT_3_/FT_4_ levels did not significantly correlate with Ca, P, CK, and CK-MB (*p*>0.05) (**Supplementary Table 1**). Binary logistic regression analysis was performed with whether fatigue occurred in patients with COVID-19 as the dependent variable and thyroid hormone level as the independent variable, and the results showed that FT_3_ level was negatively correlated with the occurrence of fatigue in patients with COVID-19, and that low T_3_ was a risk factor for the occurrence of fatigue in patients with COVID-19 (*p*<0.05). After correcting for confounding factors such as age, gender, Ca, and P, FT_3_ was negatively correlated with the occurrence of fatigue in patients with COVID-19, suggesting that low FT_3_ was a risk factor for the occurrence of fatigue in patients with COVID-19 (*p*<0.05) (**Table 3**). There was no statistically difference between TSH, FT_4_, and FT_3_/FT_4_ and the occurrence of fatigue in patients with COVID-19 (*p*>0.05) (**Supplementary Table 2**).

**Table 2:** Results of correlation analysis of features associated with fatigue scores.

	*r*	*p*
TSH	0.022	0.893
FT_3_	-0.251	0.119
FT_4_	0.076	0.642
FT3/FT4	-0.329	0.038*
Ca	0.173	0.286
P	-0.045	0.782
CK	-0.077	0.647
ALT	0.055	0.737
AST	0.018	0.91
SPINA-GD	-0.369	0.019*
TSHI	-0.146	0.370
FT4/TSH	-0.014	0.932

TSH, thyroid stimulating hormone; FT_3_, serum free triiodothyronine; FT_4_, serum free thyroxine; ALT, alanine aminotransferase; AST, aspartate aminotransferase; ALP, alkaline phosphatase; Ca, Calcium; CK, creatine phosphokinase; P, phosphorus; PCT, procalcitonin; CRP, c-reactive protein; SPINA-GD, the sum activity of deiodinases; SPINA-GT, the secretory capacity of the thyroid gland; TSHI, thyroid stimulating hormone index.

*Level of significance p<0.05.

**Table 3:** Associated FT_3_ related to fatigue by multivariate binary logistic analysis in COVID- 19 patients.

	B	S.E	Wald	*p*	*OR*	95%*CI*
FT_3_	-1.358	0.639	4.510	0.034*	0.257	0.074 0.901
Age	0.003	0.021	0.023	0.880	1.003	0.962 1.046
sex	0.583	0.777	0.563	0.453	1.791	0.391 8.211
Ca	0.190	2.284	0.007	0.934	1.209	0.014 106.375
P	-0.608	0.972	0.391	0.532	0.544	0.081 3.661
FT_3_*	-1.491	0.712	4.387	0.036**	0.225	0.056 0.909

*adjusted for age, sex, calcium and phosphorus.

**Level of significance p<0.05.

### 3.3 Predictive value of FT_3_ levels in fatigue in COVID-19 patients

**Figure 2:**
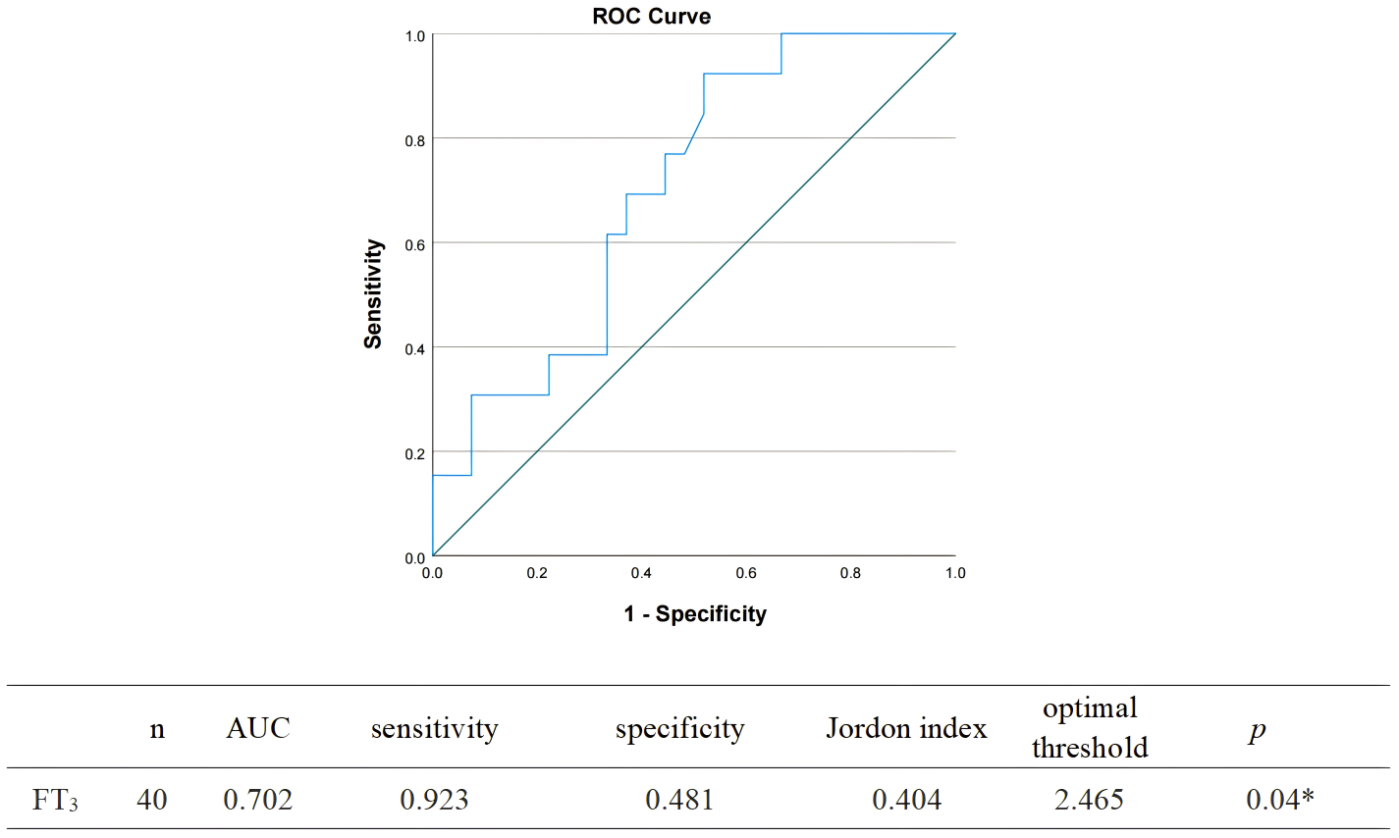
Receiver operating characteristic curve analysis determining the optimal threshold of FT_3_ for predicting fatigue after 6 months of COVID-19 infection.

The ROC curve was plotted with FT_3_ as the test variable and whether fatigue occurred in patients with COVID-19 as the state variable, and the results showed that the AUC of FT_3_ level for predicting the occurrence of fatigue in patients with COVID-19 was 0.702 (95% *CI* 0.54-0.87, *p*<0.05). Based on the maximum of the Jordon index as the critical value, when FT_3_ took a value less than 2.47 pmol/L, it was the best critical value to predict the occurrence of fatigue in patients with COVID-19, with a sensitivity of 92.3% and a specificity of 51.9% (**Figure 2**).

## 4 Discussion

COVID-19 can affect multiple systems throughout the body. As an important organ of the endocrine system, the thyroid gland is directly or indirectly affected by COVID-19. The effect of COVID-19 on the thyroid gland may be caused by directly (caused by direct cytotoxicity of the virus) or indirectly (caused by abnormal immune inflammatory response to the virus, which may involve coagulation, cytokines and complement systems) (9). NTIs is common in COVID-19 patients. A retrospective cohort study of Zou et al. included 149 COVID-19 patients, of which 41 (27.52%) were diagnosed with NTIs (10). A prospective study included 196 patients diagnosed with COVID-19 and found that 60% of ICU patients and 36% of general ward patients showed NTIs (11). In our study, 57.5% of all follow-up patients showed NTIs.

The main manifestation of NTIs is the decrease in T_3_ level. The mechanism includes the change of half-life of thyroid hormone in circulation, the change of cell sensitivity to thyroid hormone, the change of tissue uptake of thyroid hormone and the change of deiodinase activity which converts thyroid hormone into active and inactive forms respectively (12). After T_4_ deiodination, plasma T_3_ is mainly derived from the role of DIO1 in the liver. During NTIs, the activity of DIO1 in the liver and DIO2 in skeletal muscle decreased, and the activity of DIO3 in the liver and skeletal muscle increased (13). This may be a physiological adaptation to reduce energy needs during acute diseases. Under high energy demand conditions such as diseases, skeletal muscle is the main organ responsible for glucose uptake in response to insulin. In this case, muscle protein catabolism can be stimulated to maintain energy in other organs, and a decrease in T_3_ levels reduces metabolic activity and reduces energy loss in these patients (14–17).

After the recovery of COVID-19, some people still suffer from persistent and periodic symptoms. A Chinese study that included 1733 patients who had been hospitalized for COVID-19 found that at least one skeletal muscle-related symptom was still present in 76% of patients at 6 months, with fatigue or muscle weakness accounting for 63% of these (18). An Italian study reported fatigue as the predominant symptom (53.1%) of persistent symptoms 60 days after a COVID-19 infection (19). A meta-analysis showed that fatigue was the most common symptom of long covid syndrome (28.4%). Even in the non-hospitalized population, the most common long covid syndrome symptom is still fatigue, which is present in 34.8% (20). Therefore, chronic fatigue syndrome (CFS) is the most common complaint of patients. CFS, also known as myalgia encephalomyelitis, is a complex heterogeneous disorder characterized by disabling fatigue, cognitive impairment, sleep disruption, and accompanying bone and muscle pain that persists for more than 6 months and does not improve with rest (21, 22). The global pooled prevalence of CFS among long covid syndrome patients is 45.2% (23). A meta-analysis showed that the total prevalence reported by Western and Asian populations was comparable (1.32 ± 1.45% vs 1.51 ± 1.74) (24). A prospective study showed that 75.9% of patients reported fatigue 3-6 months after infection with COVID-19, which was consistent with our study (5). In our study, 70% of patients were still fatigued 6 months after infection with COVID-19. The prevalence of CFS has been reported to be 2-3 times higher in females than in males, but there was no significant gender difference in this study (25). A retrospective study showed that 48.9% of CFS patients diagnosed after COVID-19 infection were male and 51.1% were female (26). We speculate that this may be due to the fact that infections are a significant trigger for the development of CFS, and that infections make the gender difference negligible.

CFS may be associated with changes in hormone levels in the hypothalamus-pituitary-thyroid axis (27). Studies have shown that thyroid hormone function in CFS is similar to NTIs. A Serbian study found that compared with the healthy group, although the basal serum T_3_ of all subjects was normal, the T_3_ concentration in the CFS group was significantly reduced (28). In this study, the level of FT_3_ in the fatigue group was significantly lower than that in the non-fatigue group (2.52 ± 0.63 vs 3.00 ± 0.56 *p*< 0.05), and 64.3% of the fatigue group showed NTIs. FT_3_/FT_4_ is commonly used to evaluate the sensitivity of peripheral thyroid, which reflects 5’-deiodinase activity. SPINA-GD represents the total activity of peripheral deiodinase. This study shows that although there is no statistical difference in SPINA-GD between the two groups, SPINA-GD in the fatigue group is lower than that in the non-fatigue group. Correlation analysis shows that FT_3_/FT_4_ and SPINA-GD are related to fatigue (*p*<0.05), which indicates that fatigue in patients with COVID-19 may be related to subtle changes in deiodinase. Although NTIs may be beneficial in the acute phase of critical illness, it may hinder the recovery of patients in the case of long-term critical illness (29). Low T_3_ may affect brain tissue perfusion and energy metabolism, leading to fatigue (30). This study shows that low T_3_ is a risk factor for fatigue. When FT_3_ value is less than 2.47 pmol/L, it is the best critical value to predict fatigue in COVID-19 patient.

Even though the study has provided interesting findings, it had some few limitations. Certain limitations of the study are as follows: Firstly, the sample size is relatively small, resulting in a higher number of patients lost to follow-up, which may introduce a bias of loss of follow-up. Secondly. The patients included were all Chinese, potentially limiting the generalizability of the findings to other regions or ethnic groups. Thirdly, the thyroid hormone levels of COVID-19 patients after 6 months were not measured again, potentially limiting the understanding of the long-term effects of thyroid hormones on CFS. Fourthly, the average age of both groups is over 60 years old, and there is a lack of data on the young population. Finally, a meta study showed that the prevalence of CFS was 0.10% based on the physician diagnosis, and the prevalence of CFS was 2.03% in the questionnaire interview without medical testing (24). Therefore, the prevalence of CFS based on the questionnaire survey in our study may be overestimated. In the future, large-scale prospective studies are needed to further explore the mechanism of thyroid hormones affecting CFS after COVID-19.

Furthermore, in addition to thyroid hormones, symptoms of chronic fatigue may also be caused by damage to multiple organ systems during COVID-19. The occurrence of CFS in COVID-19 may be related to excessive anti-inflammatory response, and the dominant disease pattern of alternatively polarized macrophage cells directly induces the occurrence of CFS (31). Hypothalamic-pituitary-adrenal (HPA) axis disturbances have also been found in CFS, which are manifested by mild cortisol decrease, enhanced negative feedback, and a blunted response to excitation (32). COVID-19 can also cause skeletal muscle damage resulting from changes in the structure and function of the neuromuscular system through mechanisms such as direct infiltration, inflammatory response, hypoxia and other mechanisms to cause skeletal muscle damage, resulting in fatigue (33). A study reported that the immune system of ME/CFS patients produces an imbalanced ratio of pro-inflammatory and anti-inflammatory cytokines in the early stages of the disease (34). But CRP and PCT are not statistically significant in our study. In conclusion, the occurrence of CFS in COVID-19 patients is not a role of a single hormonal axis, but a combination of factors.

## 5 Conclusions

In summary, this study demonstrates that the majority of patients still experience fatigue six months after contracting the COVID-19 infection. The level of FT_3_ in the fatigue group was lower, suggesting a potential correlation between FT_3_ and the persistence of fatigue following COVID-19 infection. The mechanism of fatigue may be related to subtle changes in the hypothalamus-pituitary-thyroid axis and deiodinase. In addition, this study found that low T_3_ is a risk factor for fatigue. When the value of FT_3_ is less than 2.47 pmol/L, it serves as the optimal critical threshold to predict fatigue in patients with COVID-19. In the future, prospective studies are needed to confirm whether monitoring serum FT_3_ level can predict the occurrence of CFS in patients with COVID-19, and then help to improve the symptoms of patients.

## Data Availability

The original contributions presented in the study are included in the article/**Supplementary Material**. Further inquiries can be directed to the corresponding authors.

## References

[1] Scappaticcio L Pitoia F Esposito K Piccardo A Trimboli P. Impact of COVID-19 on the thyroid gland: an update. Rev Endocr Metab Disord. (2021) 22:803–15. doi: 10.1007/s11154-020-09615-z PMC7688298 33241508

[2] Feng W Zongxun Y. Study on the relationship between thyroid hormone level and post-stroke fatigue. Chin J Med Guide. (2020) 22:544–8. doi: 10.12083/SYSJ.2020.14.277

[3] Zhang Y Lin F Tu W Zhang J Choudhry AA Ahmed O. Thyroid dysfunction may be associated with poor outcomes in patients with COVID-19. Mol Cell Endocrinol. (2021) 521:111097. doi: 10.1016/j.mce.2020.111097 33278491 PMC7709789

[4] Szczerbiński Ł Okruszko MA Szabłowski M Sołomacha S Sowa P Kiszkiel Ł. Long-term effects of COVID-19 on the endocrine system - a pilot case-control study. Front Endocrinol (Lausanne). (2023) 14:1192174. doi: 10.3389/fendo.2023.1192174 37790604 PMC10544976

[5] Cornelissen MEB Bloemsma LD Vaes AW Baalbaki N Deng Q Beijers RJHCG. Fatigue and symptom-based clusters in post COVID-19 patients: a multicenter, prospective, observational cohort study. J Transl Med. (2024) 22:191. doi: 10.1186/s12967-024-04979-1 38383493 PMC10880228

[6] Chalder T Berelowitz G Pawlikowska T Watts L Wessely S Wright D. Development of a fatigue scale. J Psychosom Res. (1993) 37:147–53. doi: 10.1016/0022-3999(93)90081-P 8463991

[7] Hewlett S Dures E Almeida C. Measures of fatigue: Bristol Rheumatoid Arthritis Fatigue Multi-Dimensional Questionnaire (BRAF MDQ), Bristol Rheumatoid Arthritis Fatigue Numerical Rating Scales (BRAF NRS) for severity, effect, and coping, Chalder Fatigue Questionnaire (CFQ), Checklist Individual Strength (CIS20R and CIS8R), Fatigue Severity Scale (FSS), Functional Assessment Chronic Illness Therapy (Fatigue) (FACIT-F), Multi-Dimensional Assessment of Fatigue (MAF), Multi-Dimensional Fatigue Inventory (MFI), Pediatric Quality Of Life (PedsQL) Multi-Dimensional Fatigue Scale, Profile of Fatigue (ProF), Short Form 36 Vitality Subscale (SF-36 VT), and Visual Analog Scales (VAS). Arthritis Care Res (Hoboken). (2011) 63 Suppl 11:S263–S86. doi: 10.1002/acr.20579 22588750

[8] Xuelin T Ji G Yan Z Yingchun Z. Research progress on the application of fatigue scale (FS-14). Chin Gen Pract Nurs. (2022) 20:2193–7. doi: 10.12104/j.issn.1674-4748.2022.16.008

[9] Ruiz-Núñez B Tarasse R Vogelaar EF Janneke Dijck-Brouwer DA Muskiet FAJ. Higher prevalence of “Low T3 syndrome” in patients with chronic fatigue syndrome: A case–control study. Front Endocrinol. (2018) 9:97. doi: 10.3389/fendo.2018.00097 PMC5869352 29615976

[10] Zou R Wu C Zhang S Wang G Zhang Q Yu B. Euthyroid sick syndrome in patients with COVID-19. Front Endocrinol. (2020) 11:566439. doi: 10.3389/fendo.2020.566439 PMC7575767 33117282

[11] Vassiliadi DA Ilias I Pratikaki M Jahaj E Vassiliou AG Detsika M. Thyroid hormone alterations in critically and non-critically ill patients with SARS-CoV-2 infection. Endocrine Connections. (2021) 10:646–55. doi: 10.1530/EC-21-0029 PMC8240704 34010152

[12] Stanculescu D Bergquist J. Perspective: drawing on findings from critical illness to explain myalgic encephalomyelitis/chronic fatigue syndrome. Front Med. (2022) 9:818728. doi: 10.3389/fmed.2022.818728 PMC8957276 35345768

[13] Lado-Abeal J Romero A Castro-Piedras I Rodriguez-Perez A Alvarez-Escudero J. Thyroid hormone receptors are down-regulated in skeletal muscle of patients with non-thyroidal illness syndrome secondary to non-septic shock. Eur J Endocrinol. (2010) 163:765–73. doi: 10.1530/EJE-10-0376 20736347

[14] Argilés JM Campos N Lopez-Pedrosa JM Rueda R Rodriguez-Mañas L. Skeletal muscle regulates metabolism via interorgan crosstalk: roles in health and disease. J Am Med Directors Assoc. (2016) 17:789–96. doi: 10.1016/j.jamda.2016.04.019 27324808

[15] Van den Berghe G. On the neuroendocrinopathy of critical illness. Perspectives for feeding and novel treatments. Am J Respir Crit Care Med. (2016) 194:1337–48. doi: 10.1164/rccm.201607-1516CI 27611700

[16] Fliers E Bianco AC Langouche L Boelen A. Thyroid function in critically ill patients. Lancet Diabetes Endocrinol. (2015) 3:816–25. doi: 10.1016/S2213-8587(15)00225-9 PMC4979220 26071885

[17] Bloise FF Cordeiro A Ortiga-Carvalho TM. Role of thyroid hormone in skeletal muscle physiology. J Endocrinol. (2018) 236:R57–68. doi: 10.1530/JOE-16-0611 29051191

[18] Huang C Huang L Wang Y Li X Ren L Gu X. RETRACTED: 6-month consequences of COVID-19 in patients discharged from hospital: a cohort study. Lancet. (2021) 397:220–32. doi: 10.1016/S0140-6736(20)32656-8 PMC7833295 33428867

[19] Carfì A Bernabei R Landi F. Persistent symptoms in patients after acute COVID-19. Jama. (2020) 324(6):603–605. doi: 10.1001/jama.2020.12603 PMC7349096 32644129

[20] O’Mahoney LL Routen A Gillies C Ekezie W Welford A Zhang A. The prevalence and long-term health effects of Long Covid among hospitalized and non-hospitalized populations: a systematic review and meta-analysis. eClinicalMedicine. (2023) 55:101762. doi: 10.1016/j.eclinm.2022.101762 36474804 PMC9714474

[21] Holmes GP Kaplan JE Gantz NM Komaroff AL Schonberger LB Straus SE. Chronic fatigue syndrome: a working case definition. Ann Intern Med. (1988) 108:387–9. doi: 10.7326/0003-4819-108-3-387 2829679

[22] Fukuda K Straus SE Hickie I Sharpe MC Dobbins JG Komaroff A. The chronic fatigue syndrome: a comprehensive approach to its definition and study. International Chronic Fatigue Syndrome Study Group. Ann Intern Med. (1994) 121:953–9. doi: 10.7326/0003-4819-121-12-199412150-00009 7978722

[23] Salari N Khodayari Y Hosseinian-Far A Zarei H Rasoulpoor S Akbari H. Global prevalence of chronic fatigue syndrome among long COVID-19 patients: A systematic review and meta-analysis. BioPsychoSocial Med. (2022) 16(1):21. doi: 10.1186/s13030-022-00250-5 PMC9589726 36274177

[24] Lim EJ Ahn YC Jang ES Lee SW Lee SH Son CG. Systematic review and meta-analysis of the prevalence of chronic fatigue syndrome/myalgic encephalomyelitis (CFS/ME). J Transl Med. (2020) 18:100. doi: 10.1186/s12967-020-02269-0 32093722 PMC7038594

[25] Arron HE Marsh BD Kell DB Khan MA Jaeger BR Pretorius E. Myalgic Encephalomyelitis/Chronic Fatigue Syndrome: the biology of a neglected disease. Front Immunol. (2024) 15:1386607. doi: 10.3389/fimmu.2024.1386607 38887284 PMC11180809

[26] Tokumasu K Honda H Sunada N Sakurada Y Matsuda Y Yamamoto K. Clinical characteristics of myalgic encephalomyelitis/chronic fatigue syndrome (ME/CFS) diagnosed in patients with long COVID. Medicina (Kaunas). (2022) 58:850. doi: 10.3390/medicina58070850 35888568 PMC9325226

[27] Fuite J Vernon SD Broderick G. Neuroendocrine and immune network re-modeling in chronic fatigue syndrome: An exploratory analysis. Genomics. (2008) 92:393–9. doi: 10.1016/j.ygeno.2008.08.008 18775774

[28] Tomic S Brkic S Lendak D Maric D Medic Stojanoska M Novakov Mikic A. Neuroendocrine disorder in chronic fatigue syndrome. Turkish J Med Sci. (2017) 47:1097–103. doi: 10.3906/sag-1601-110 29154201

[29] Boelen A Kwakkel J Fliers E. Beyond low plasma T3: local thyroid hormone metabolism during inflammation and infection. Endocrine Rev. (2011) 32:670–93. doi: 10.1210/er.2011-0007 21791567

[30] Min L Hao L Youming W Mengge Z. Study on the relationship between thyroid hormone level and post-stroke fatigue. Chin J Pract Nervous Dis. (2020) 23:1221–4. doi: 10.12083/SYSJ.2020.14.277

[31] Kovarik JJ Bileck A Hagn G Meier-Menches SM Frey T Kaempf A. A multi-omics based anti-inflammatory immune signature characterizes long COVID-19 syndrome. iScience. (2023) 26(1):105717. doi: 10.1016/j.isci.2022.105717 36507225 PMC9719844

[32] Cleare AJ. The HPA axis and the genesis of chronic fatigue syndrome. Trends Endocrinol Metab. (2004) 15:55–9. doi: 10.1016/j.tem.2003.12.002 15036250

[33] Soares MN Eggelbusch M Naddaf E Gerrits KHL van der Schaaf M van den Borst B. Skeletal muscle alterations in patients with acute Covid-19 and post-acute sequelae of Covid-19. J Cachexia Sarcopenia Muscle. (2022) 13:11–22. doi: 10.1002/jcsm.12896 34997689 PMC8818659

[34] Hornig M Montoya JG Klimas NG Levine S Felsenstein D Bateman L. Distinct plasma immune signatures in ME/CFS are present early in the course of illness. Sci Adv. (2015) 1(1):e1400121. doi: 10.1126/sciadv.1400121 26079000 PMC4465185

