# Supplementary material for: The relation between FT3 and long-term fatigue in patients with COVID-19
Source: Front Endocrinol (Lausanne). 2024 Aug 23;15:1411262. doi: 10.3389/fendo.2024.1411262 (PMC11377235; doi:10.3389/fendo.2024.1411262)
Supplement: Supplementary file 1 [file Table1.docx]

# Supplemental Table 1. Results of correlation analysis of features associated with FT3/FT4

|  | *r* | p |
| --- | --- | --- |
| P | -0.026 | 0.874 |
| Ca | 0.243 | 0.131 |
| CK | 0.248 | 0.133 |
| ALT | 0.104 | 0.523 |
| AST | -0.100 | 0.538 |
| ALP | -0.176 | 0.277 |
| MYO | -0.222 | 0.215 |
| hs-TnI | -0.409 | 0.009* |
| CK-MB | -0.209 | 0.196 |

Ca, Calcium; P, phosphorus; CK,creatine phosphokinase; ALT, alanine aminotransferase, AST, aspartate aminotransferase; ALP, alkaline

phosphatase; MYO, myoglobin; hs-TnI, high-sensitivity troponin; CK-MB, creatine kinase isoenzyme;

*Level of significance p<0.05.

Supplemental Table 2. Associated TSH, FT_4_ and FT_3_/FT_4_ related to fatigue by multivariate binary logistic analysis in COVID- 19 patients.

|  | B | S.E | Wald | *p* | *OR* | 95%*CI* |
| --- | --- | --- | --- | --- | --- | --- |
| TSH | 0.411 | 0.334 | 1.518 | 0.218 | 1.508 | 0.784-2.899 |
| FT_4_ | -0.053 | 0.117 | 0.207 | 0.649 | 0.948 | 0.754-1.192 |
| FT_3_/FT_4_ | -16.09 | 10.26 | 2.459 | 0.117 | 0.000 | 0-231.671 |

TSH, thyroid stimulating hormone; FT_3_, serum free triiodothyronine; FT_4_, serum free thyroxine.

Level of significance *p*<0.05
